# White matter disconnection and decreased functional connectivity between orbitofrontal cortex and the contralateral temporo-occipital cortex in adults with obsessive compulsive disorder

**DOI:** 10.1192/j.eurpsy.2021.375

**Published:** 2021-08-13

**Authors:** J. Queiroz, J. Oliveira, A. Maia, C. Fonseca, T. Quendera, A. Oliveira-Maia, B. Barahona-Correa

**Affiliations:** 1 Neuropsychiatry Unit, Champalimaud Research, Champalimaud Foundation Centre for the Unknown, Lisbon, Portugal; 2 Neuropsychiatry Unit, Champalimaud Research and Clinical Centre, Champalimaud Foundation Centre for the Unknown, Lisbon, Portugal; 3 Department Of Physics, Faculdade de Ciências da Universidade de Lisboa, Lisbon, Portugal; 4 Departamento De Psiquiatria E Saúde Mental, NOVA Medical School | Faculdade de Ciências Médicas de Lisboa, Lisbon, Portugal

**Keywords:** DTI, ocd, connectivity, orbitofrontal

## Abstract

**Introduction:**

Obsessive compulsive disorder (OCD) affects 2-3% of the general population. The neurobiology of OCD has been linked to dysfunction of cortico-striatal circuits connecting the orbitofrontal (OFC) to the striatum. Recently, this loop has become an approved target for non-invasive neuromodulatory treatment of OCD.

**Objectives:**

To explore structural and functional connectivity of the OFC in OCD subjects and healthy controls.

**Methods:**

14 OCD patients and 12 age/sex-matched controls underwent magnetic resonance imaging (MRI) (3T-Philips scanner) for diffusion tensor imaging (DTI) and resting state functional connectivity (rsFC). DTI images were brain extracted and corrected for movement and eddy currents. A diffusion tensor model was fitted to each voxel and used to generate Fractional Anisotropy (FA) maps. Voxel-wise statistical analysis of FA was performed using Tract-Based Spatial Statistics. RsFC images were preprocessed and seed-based correlation (SBC) analysis was performed using Data Processing Assistant for Resting-State fMRI.

**Results:**

We found decreased values of FA in the body of the Corpus Callosum bilaterally (MNI_coordinates: x= 16, y= -16, z= 33 and x= -19, y= -16, z= 42) and left superior longitudinal fasciculus in OCD patients (fig 1, left), as well as decreased rsFC of the right superior orbitofrontal seed with the left inferior frontal gyrus and left middle occipital gyrus (fig 2, right).

**Figure:**
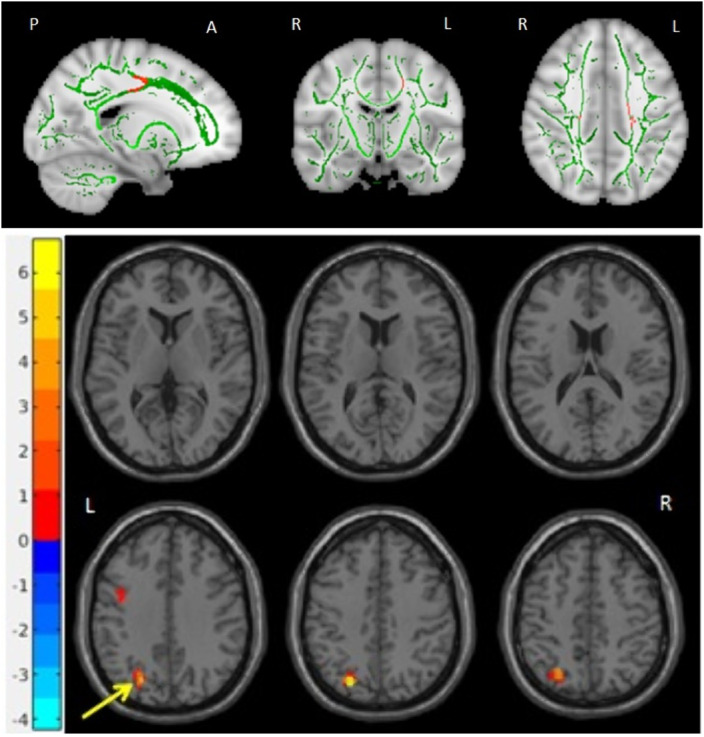

**Conclusions:**

Using an exploratory multimodal approach we found evidence of abnormal structural and functional long-range connectivity of the OFC in OCD. If confirmed in a larger sample these connectivity abnormalities could be explored as potential predictors of response to OFC-targeted non-invasive neuromodulatory interventions.

**Disclosure:**

No significant relationships.

